# Status and Barriers of Physical Activity and Exercise in Community-Dwelling Stroke Patients in South Korea: A Survey-Based Study

**DOI:** 10.3390/healthcare12060697

**Published:** 2024-03-20

**Authors:** Jung-Lim Lee, Yuna Kim, Sungchul Huh, Yong-Il Shin, Sung-Hwa Ko

**Affiliations:** 1 Research Institute for Convergence of Biomedical Science and Technology, Pusan National University Yangsan Hospital, Yangsan 50612, Republic of Korea; lim6668@naver.com (J.-L.L.); kyoona1289@gmail.com (Y.K.); dr.huhsc@gmail.com (S.H.); rmshin01@gmail.com (Y.-I.S.); 2 Department of Rehabilitation Medicine, Pusan National University Yangsan Hospital, Yangsan 50612, Republic of Korea; 3 Department of Rehabilitation Medicine, Pusan National University School of Medicine, Yangsan 50612, Republic of Korea

**Keywords:** stroke, barrier, physical activity, community-based exercise, quality of life, social participation

## Abstract

This study aimed to examine the physical activity and exercise status of stroke patients in the community after discharge and the need for community-based exercises. This study included 100 community-dwelling patients with stroke in South Korea. The survey investigated the self-assessment of health status and physical activity, demand for community-based exercise after discharge, quality of life, and social participation. Overall, 96% of the respondents recognized the need to exercise, and two-thirds exercised. The third who did not exercise cited disability (29%), lack of facilities (22%), and health concerns (13%); only 21% of participants had ever used a community exercise facility, and their satisfaction with the facility was low. The main reasons for not using community exercise facilities were concerns about accidents during exercise and accessibility issues. Among real-world community stroke patients, those who exercised regularly had higher EuroQol-5D and reintegration to normal living indices than those who did not exercise (*p* < 0.05). Although community-dwelling stroke patients were highly aware of the need for physical activity and exercise, few engaged in adequate exercise. This lack of engagement is directly linked to identifiable personal and socio-structural barriers. Addressing these barriers will improve the quality of life and social participation of patients with stroke.

## 1. Introduction

Stroke is a leading cause of death worldwide; it significantly reduces the ability of individuals to perform activities of daily living (ADL), thereby making it difficult for them to lead independent lives [1,2]. Approximately 65% of stroke patients develop serious disabilities that require assistance in daily activities after onset, necessitating long-term rehabilitation [3]. 

After discharge, stroke patients require physical activity and exercise to reduce their disabilities and improve their overall functional abilities [4]. Most exercises are conducted in hospitals; however, there is increasing interest in community-based group programs for physical activity and exercise [5]. This shift in focus from hospital to community settings is crucial given the growing prevalence of stroke and the increasing need for sustainable long-term rehabilitation solutions. 

Physical activity and exercise after discharge in stroke patients are known to have a positive impact on improving the quality of life (QoL), reducing stroke mortality and recurrence rates, and enhancing physical, cognitive, and psychological functions [6,7,8,9]. Additionally, community-based exercise has been shown to positively affect the QoL and social participation of patients [10,11]. Therefore, stroke guidelines strongly recommend moderate physical activity and exercise for 30–60 min, 4–7 days a week, along with activities of daily living for the secondary prevention of stroke [12,13,14]. However, previous studies have revealed that the compliance rate for aerobic exercise among non-disabled stroke survivors is lower than that among healthy controls and that they tend to have longer periods of sedentary behavior [15,16,17]. Moreover, the average daily step count of patients with chronic stroke is only approximately half that of a healthy individual and is significantly lower than that of patients with other diseases [18,19,20]. 

Despite these known advantages of exercise and physical activity, the current literature indicates a significant gap in understanding and addressing the specific barriers to physical activity and exercise among community-dwelling stroke survivors. Numerous studies have shown that community-dwelling patients with stroke have reduced physical activity and exercise [15,16,17,18]. However, research on why these patients limit their physical activity and exercise is lacking. In a systematic review of six studies, the most commonly reported barriers were lack of motivation, environmental factors (e.g., transportation), health issues, and stroke disability; however, the total number of studies was small [21]. 

This study investigated the exercise practices of community-dwelling stroke patients after hospital discharge, identified barriers to their participation, and assessed the demand for community-based exercises in South Korea. Additionally, we aimed to determine the impact of exercise on the QoL and social participation of patients in the real world. Thus, this study aimed to address the notable absence of research focusing on barriers to physical activity among community-dwelling stroke survivors, contributing valuable insights that could inform the design of targeted community-based rehabilitation interventions.

## 2. Materials and Methods

### 2.1. Subjects

A survey was conducted from November 2021 to November 2022. Inclusion criteria for the study were patients aged 18 and over, community-dwelling stroke survivors attending the outpatient clinic at Pusan National University Yangsan Hospital (Yangsan-si, Gyeongsangnam-do, Republic of Korea) for post-stroke complications or secondary prevention management, specifically those whose stroke onset was more than 6 months ago. Exclusion criteria included patients with cognitive impairments, those unable to comprehend the study’s content or respond to the survey, and individuals who did not provide consent, either personally or through their guardians. All participants provided informed consent prior to the survey. 

This study was approved by the Clinical Review Committee of Yangsan Pusan National University Hospital (IRB No. 04-2021-050), and all participants and their representatives provided written informed consent.

### 2.2. Survey

The cross-sectional survey was conducted using structured questionnaires and face-to-face interviews with stroke patients. The survey investigated the patients’ basic information, self-assessment of health status and physical activity, demand for community-based exercise after discharge, QoL, and social participation (Table 1). Information on stroke type and onset time was collected from medical records according to the personal information disclosure consent form included in the subject description and consent form. Self-assessment of health status and physical activity after discharge investigated the current perception of health status and exercise needs, current exercise status, exercise intensity and frequency, and reasons for not exercising. The demand for community-based exercise after discharge included questions about the use and satisfaction with local exercise facilities, reasons for not using them, and essential elements of community-based exercise for stroke patients. Post-discharge QoL and social participation were evaluated using the EuroQol-5D (EQ-5D) and Reintegration to Normal Living Index (RNLI). 

#### 2.2.1. EuroQol-5D (EQ-5D)

To assess participants’ QoL, the study used the EuroQol 5 Dimension 3 Level (EQ-5D-3L) instrument. This tool assesses QoL in five dimensions: mobility, self-care, usual activities, pain/discomfort, and anxiety/depression. For each dimension, participants’ experiences are categorized using a three-level Likert scale indicating the presence of “no problems,” “moderate problems,” or “severe problems” [22]. In conducting this analysis, the EQ-5D index scores, which are indicative of subjects’ QoL, were calculated based on a scoring framework according to established guidelines [23]. This framework assesses individual experiences across five essential health dimensions, namely:

- Mobility (M): ‘Slight’ issues were assigned a value of M2 = 1, and ‘severe’ issues, M3 = 1.
- Self-Care (SC): For ‘slight’ and ‘severe’ issues, values of SC2 = 1 and SC3 = 1 were respectively applied.
- Usual Activities (UA): ‘Slight’ and ‘severe’ difficulties received scores of UA2 = 1 and UA3 = 1, respectively.
- Pain/Discomfort (PD): PD2 = 1 was allocated for ‘slight’ discomfort, and PD3 = 1 for ‘severe’.
- Anxiety/Depression (AD): ‘Slight’ issues were quantified as AD2 = 1, and ‘severe’ as AD3 = 1.
- No. of severe problem (N): If there is at least one dimension in which severe problems are reported, N3 = 1 is applied.

The resultant EQ-5D index is thereby formulated as: (y = 1 − (0.050 + 0.096 × M2 + 0.418 × M3 + 0.046 × SC2 + 0.136 × SC3 + 0.051 × UA2+ 0.208 × UA3 + 0.037 × PD2 + 0.151 × PD3 + 0.043 × AD2 + 0.158 × AD3 + 0.050 × N3 [23]. According to a systematic review of the EQ-5D, this assessment tool is considered reliable, with an Intraclass Correlation Coefficient (ICC) of 0.52~0.83, and is validated as an effective measure [24,25].

#### 2.2.2. Reintegration to Normal Living Index (RNLI)

In the current study, the Reintegration to Normal Living Index (RNLI) served as the primary instrument to quantitatively assess the extent to which participants returned to their usual level of functioning after experiencing a significant illness or injury [26]. This self-administered instrument includes 11 items that explore various facets of life affected by debilitating events, including mobility, autonomy in personal care, engagement in daily tasks, leisure activities, coping mechanisms, family roles, participation in social gatherings, maintenance of personal relationships, and self-presentation. Respondents are asked to rate their level of reintegration for each aspect on a continuum from 1 (indicating minimal reintegration) to 10 (indicating full reintegration), resulting in a cumulative score ranging from 11 to 110. For analytical purposes, these aggregate scores are then normalized to a 100-point metric (Total Score/110) ×100 [27]. According to a systematic review of the RNLI, while its validity may not match that of other assessments, its reliability is high, with an Intraclass Correlation Coefficient (ICC) of 0.87, indicating it is a trustworthy evaluation tool [28].

### 2.3. Statistical Analyses

The data collected in this study were analyzed using SPSS 22.0. Most of the survey content was expressed as a percentage of the total number of respondents. To analyze QoL and social participation according to self-exercise status, the EQ-5D index and RNLI scores were compared between the exercise and non-exercise groups using an independent sample *t*-test. Statistical significance was set at *p* < 0.05. 

## 3. Results

### 3.1. Participant Characteristics

The demographic characteristics of the participants are presented in Table 2. Regarding age, 5 participants were <40 years, 37 were between 40 and 59 years, 34 were between 60 and 69 years, and 24 were >70 years. Among them, 56 were male and 44 were female. The stroke types varied, with 43 cerebral infarctions and 57 cerebral hemorrhages. The duration of stroke onset was < 12 in 15 patients, 12–59 months in 38 patients, 60–120 months in 31 patients, and >120 months in 13 patients. Regarding functional independence, 53 patients scored 0 to 2 on the modified Rankin Scale (mRS), indicating independence, while 47 scored 3–5, signifying dependence on daily activities (Table 2). 

### 3.2. Self-Assessment of Health Status and Physical Activity Status after Discharge from Hospital

In the self-assessments of health, using a five-point scale, participants rated themselves as follows: 2 = very good, 15 = good, 41 = fair, 24 = poor, and 7 = very poor. Regarding the perceived necessity of exercise, 59%, 27%, 10%, and 4% found it very necessary, necessary, moderately necessary, and unnecessary, respectively. 

Currently, 67% of the patients exercised. Of those who exercised, 88% performed low-intensity exercises (light physical activities such as walking, stretching, or strolling), 9% engaged in moderate-intensity exercises (slightly strenuous activities such as slow swimming, badminton, or table tennis), and 3% participated in high-intensity exercises (activities that significantly increase heart rate and breathing, such as jogging, hiking, soccer, or fast swimming). Additionally, 57% of the patients reported exercising daily, 21% exercised ≥3 days a week, 15% exercised twice a week, 4% exercised once a week, and 3% exercised less than once a week. 

**Figure 1:**
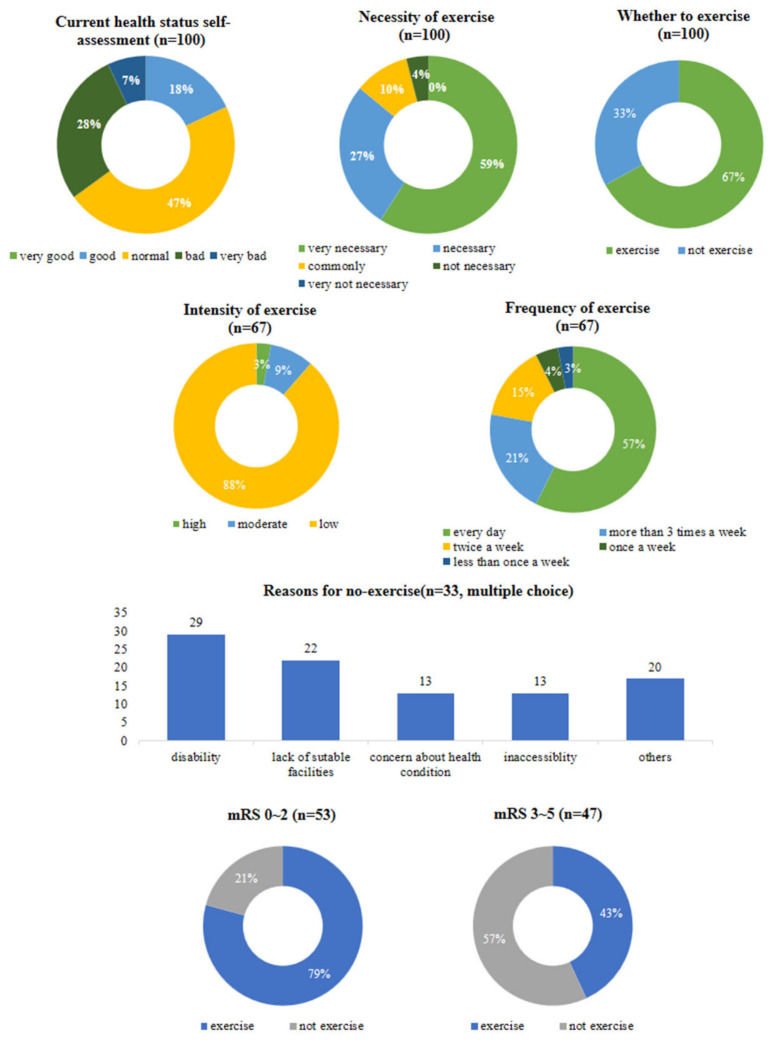
Status of health and exercise in the community-dwelling stroke patients.

However, 33% of the patients reported not exercising at all. Disability (29%) was the most common reason for not exercising, followed by a lack of appropriate facilities (22%), concerns about health conditions (13%), and inaccessibility were the reasons given for not exercising. Other barriers, such as lack of time, cost, information, and facility expertise, were also cited. Community-dwelling stroke patients who did not exercise reported their disability as the reason for not exercising. To analyze the relationship between the actual degree of disability and exercise, we conducted further analyses using the mRS to objectively assess disability levels. Patients were divided into two groups based on their mRS scores: mild disability (mRS 0–2) and moderate-to-severe disability (mRS 3–5). The analysis revealed that 21% of patients with mild disability did not exercise, compared to 57% of those with moderate-to-severe disability, indicating a higher percentage of non-exercise among patients with greater disability severity (Figure 1).

### 3.3. Demands for Community-Based Exercise after Discharge

Of the 100 respondents, 21% had used a community exercise facility, compared to 79% who had not. Among the participants who used the facility, 19% were satisfied, 24% were neutral, 38% were dissatisfied, and 19% were extremely dissatisfied. When we asked the 79 respondents who had never used a facility why they did not or could not use one, concerns about their health and accidents while exercising were the most common reasons (47%), followed by inaccessibility (23%). Twenty-one percent of the participants cited a lack of facilities. 

**Figure 2:**
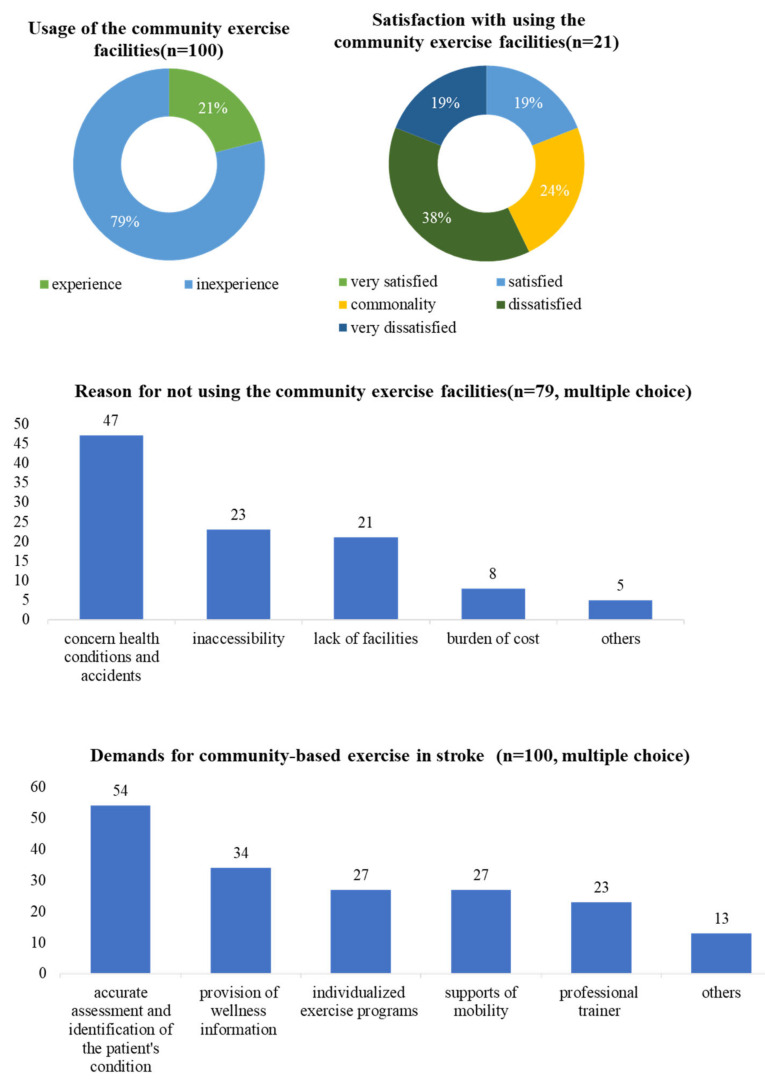
Status regarding the use of and demand for community exercise facilities for stroke patients.

When all respondents were asked to identify the essential elements needed for community-based exercise, the most popular response was accurate assessment and diagnosis of patient condition (30%), followed by the provision of wellness information (19%). Support for individualized exercise programs (15%) and mobility (15%) were the next most popular responses. The need for a professional trainer was also mentioned, as was the need for a stepwise exercise plan (Figure 2). 

### 3.4. QoL and Social Participation According to Exercise after Discharge

The mean EQ-5D index score for all subjects was 0.65 ± 0.24. The average RNLI score for social engagement was 46.5 ± 28.24. 

There were 67 and 33 participants in the exercise and non-exercise groups, respectively, based on whether they had exercised in the past 3 months. The EQ-5D index was significantly higher in the exercise group (0.73 ± 0.20) than in the non-exercise group (0.49 ± 0.24; *p* < 0.001). Furthermore, the RNLI was significantly higher in the exercise group (56.8 ± 27.5) than in the non-exercise group (25.6 ± 15.5; *p* < 0.001).

**Figure 3:**
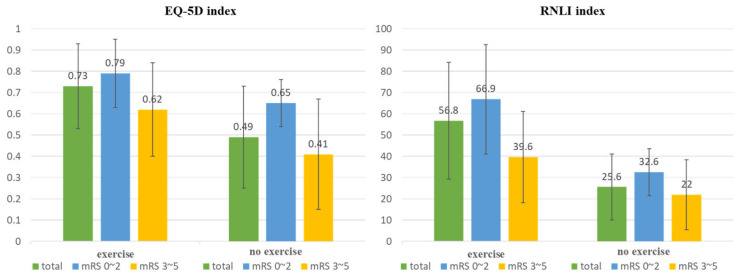
EuroQol-5D (EQ-5D) and Reintegration to Normal Living Index (RNLI) according to community-based exercise.

A subgroup analysis was conducted by dividing the participants into those with mild disability (mRS 0–2) and those with moderate-to-severe disability (mRS 3–5). The exercise group with mRS 0–2 had an EQ-5D index of 0.79 ± 0.16, which was significantly higher than the non-exercise group (0.65 ± 0.11; *p* = 0.009). The exercise group also had a significantly higher RNLI index value of 66.9 ± 25.7 compared to the non-exercise group (32.6 ± 11.0; *p* < 0.001). In patients with an mRS score of 3–5, the exercise group had a higher EQ-5D index (0.62 ± 0.22) compared to the non-exercise group (0.41 ± 0.26), which was statistically significant (*p* = 0.004). Additionally, the RNLI index was higher in the exercise group (39.6 ± 21.5) than in the non-exercise group (22 ± 16.4), which was also statistically significant (*p* = 0.003) (Figure 3).

## 4. Discussion

In this study, we investigated the status of self-exercise and the need for community-based exercises among patients with stroke. In addition, we compared QoL and social participation according to exercise among stroke patients in the community. As a result, the status of self-exercise among community-dwelling stroke patients showed that the patient’s awareness of the need for exercise was high and that many patients engaged in self-exercise. The results of a survey on the demand for community-based exercises showed that the proportion of patients using the facility was very low, as was user satisfaction. The main reasons for not using these facilities were health concerns and accessibility issues. In the comparison analysis, the exercise group had a higher QoL and social participation than the non-exercise group. 

Community-based exercises reduce stroke mortality and recurrence rates and provide physical, cognitive, and psychological benefits [6,7,8,9]. The recommended number of steps per day for the healthy population is 10,000, and the recommended number of steps per day for people with disabilities or chronic conditions is 6500 to 8500 steps [19]. However, a systematic review of physical activity in stroke patients found that the average number of steps per day was 5535 for subacute stroke patients and 4078 for chronic stroke patients, which is less than the recommended number of steps, which becomes even less as the stroke progresses to the chronic phase [18].

Many stroke guidelines recommend at least 10 min of moderate-intensity physical activity at least four times a week or 20 min of vigorous-intensity physical activity at least twice a week [7,8,29,30,31,32]. Our study found that most patients with stroke returning to the community after hospital discharge recognized the need to exercise, and those who exercised regularly were more likely to do so than those who did not. Compared to the guidelines, our study found that about 80% of participants were exercising three or more times a week, indicating a relatively appropriate frequency of exercise in many cases. However, when it comes to exercise intensity, only 12% of participants engaged in moderate to high-intensity exercise, revealing that the intensity of exercise was lower than the recommended level. Additionally, it is noteworthy that one-third of the study population did not exercise at all. In particular, patients who did not exercise had more moderate-to-severe disabilities than mild disabilities. The reasons for this were identified as a combination of personal factors, such as disability or health concerns, and external (organizational) factors, such as the lack of suitable facilities for stroke patients to exercise and the lack of accessibility. 

A 2013 systematic review reported published studies on the perceived barriers to and motivators for physical activity after stroke [21]. This systematic review included six papers that provided data on 174 stroke survivors (ranging from 10 to 83 per paper). The results showed that the most reported barriers were lack of motivation, environmental factors (e.g., transport), health concerns, and stroke impairments. Since then, several studies have been published in each country [33,34,35]. In the barriers to activity and participation for stroke survivors’ study in China, physical/structural barriers were the most frequently cited barriers at 77.5%, followed by services/assistance barriers at 64.6%. Attitudinal/support barriers and policy barriers were the least frequently cited barriers at 28.3% and 25%, respectively [33]. A 2022 study of 30 stroke patients in Quebec, Canada, did not categorize barriers but found that fear of falling was the top barrier at 47%, followed by discomfort with exercising at the gym (33%), lack of energy (30%), and fear of injury (27%) [34]. In 2023, a Singaporean study conducted a comprehensive survey of 38 stroke survivors’ exercise barriers, categorized into different barriers: organizational barriers (associated with fitness centers), intrapersonal barriers (associated with self), community barriers (associated with the environment) and interpersonal barriers (associated with friends and family) [29]. The results showed that the most reported barriers to exercise among stroke survivors were organizational barriers (e.g., lack of accessible classes and programs at the fitness center, lack of support from fitness center staff) and intrapersonal barriers (e.g., fatigue, lack of motivation, fear of injury) [35]. While these studies differ in prioritization, most reported personal factors, such as disability and fear of falling, as the main barriers, in addition to structural issues, such as lack of exercise facilities and accessibility. Our study in South Korea showed similar results. Disability and fear of injury are the top intrapersonal barriers, and lack of exercise facilities and accessibility are the top community barriers. While there were no significant differences between the studies, we believe that realistic solutions to address the barriers identified in our study will vary based on the cultural, geographical, and welfare policy contexts of each country. Ideally, minimizing disabilities following a stroke necessitates prioritizing prompt therapeutic interventions and rehabilitation. Concurrently, developing educational initiatives aimed at reducing injury fears by sharing knowledge on safe exercise practices and initiating community-based programs that foster motivation and peer support is crucial for overcoming psychological barriers and promoting physical activity. Enhancing the availability of specially tailored exercise facilities and physical accessibility (e.g., barrier-free environments, upgrading transportation systems to accommodate diverse needs, including accessible buses, trains, and taxis) for community-dwelling stroke patients with disabilities is essential. For this, cooperation with organizations and institutions and governance efforts to improve public amenities and exercise infrastructure is vital.

Previous studies have shown that exercise positively affects the QoL and social engagement of stroke patients. A self-exercise program for 12 stroke patients was shown to improve the QoL, as measured by the Short Form Health Survey 8 scale [10,11]. Furthermore, a community-based group exercise program for 20 stroke patients was shown to have a positive effect on QoL and social participation, as measured by the Stroke Impact Scale [11]. This study also found that exercisers had a significantly better QoL, as measured by the EQ-5D, and social participation, as measured by the RNLI than non-exercisers in the community. Our study included 100 community-dwelling stroke patients, which is more than previous studies, thereby strengthening the evidence for the positive role of exercise participation after hospital discharge on QoL and social participation and emphasizing the need to encourage it. In addition, in a subgroup analysis based on the mRS, the exercise group had significantly better QoL and social participation than the non-exercise group in terms of both mild and moderate-to-severe disability. Through sub-analysis, we found that although community-dwelling stroke patients with moderate-to-severe disability are more likely to not exercise, exercising can improve their QoL and social participation just as well as those with mild disability. This finding showed the universal benefits of exercise across different levels of stroke-induced disability. However, it has been confirmed that community-dwelling stroke patients perceive ‘disability’ as the most significant barrier to community-based exercise, with actual participation rates remaining low. Therefore, to enhance the QoL and social participation among community-dwelling stroke patients, accessible exercise programs, facilities, and expert guidance tailored to the disabilities need to be offered.

This study, compared to previous research, conducted a survey with a sufficient number of participants and was the first study in South Korea. However, it has some limitations. First, there was only targeting patients within a specific region of South Korea. Second, in general, ischemic strokes are more prevalent than hemorrhagic strokes, but our study included a higher proportion of hemorrhagic stroke patients. This discrepancy may limit the representativeness of our findings to the wider stroke survivor population. Further research should aim to investigate effective strategies to promote exercise participation across the spectrum of disability severity in stroke survivors. Therefore, to address this issue comprehensively, a multicenter, large-scale, in-depth national study covering the entire country is required.

## 5. Conclusions

This study examined the exercise status of 100 community-dwelling patients with stroke, their challenges and need for access to exercise facilities, and the positive effects of exercise on their QoL and social participation in South Korea. As many stroke patients recognize the need for exercise, the many factors identified in this study need to be addressed. These include accurate diagnosis and assessment of the patient’s physical condition to address intrapersonal barriers; expansion and accessibility of appropriate facilities for stroke patients to exercise to address community barriers; consideration of organizational barriers through the dissemination of expertise on exercise in stroke patients; and social and political considerations for well-being linkages, which require approaches at the national or global level. 

## Figures and Tables

**Table 1:** Questionnaire for physical activity and exercise, and barriers in community-dwelling stroke patients.

Questions	Answers									
1. Current health status self-assessment	Very good		Good		Normal		Bad		Very bad	
2. Necessity of exercise	Very necessary		Necessary		Common		Not necessary		Very not necessary	
3. Whether to exercise	Exercise					Not exercise				
3.1.1. Intensity of exercise	High				Moderate		Low			
3.1.2. Frequency of exercise	Every day		Three or more times a week		Twice a week		Once a week		Less than once a week	
3.2. Reasons for no exercise (multiple choice)	Disability		Lack of suitable facilities		Concern about health conditions		Inadequate accessibility		Others	
4. Usage of the community exercise facilities	Experience					Inexperience				
4.1. Satisfaction with using the community exercise facilities	Very satisfied		Satisfied		Commonality		Dissatisfied		Very dissatisfied	
4.2. Reason for not using the community exercise facilities	Concern about health conditions and accidents		Difficult mobility and inadequate accessibility		Lack of time or suitable facilities		Burden of cost		Others	
5. Essential factors needed for community-based exercise (multiple choice)	Accurate assessment and identification of the patient’s condition		Providing and linking to welfare		Individual exercise program	Supports of mobility and accessibility	Qualified exercise trainers		Others	

**Table 2:** Characteristics of the study subjects.

Characteristics	(n = 100)
Age (years)	
<40	5
40–59	37
60–69	34
≥70	24
Sex	
Male	56
Female	44
Type of stroke	
Infarction	43
Hemorrhage	57
Time since stroke (months)	
<12	15
12–59	38
60–120	31
≥120	13
Modified Rankin Scale score	
0–2	53
3–5	47

## Data Availability

The dataset analyzed in the present study is not publicly available because of ethical and legal regulations regarding the protection of personal data.
